# *Lactobacillus paracasei* 259 alleviates hyperuricemia in rats by decreasing uric acid and modulating the gut microbiota

**DOI:** 10.3389/fnut.2024.1450284

**Published:** 2024-11-12

**Authors:** Chengming Bi, Lanjun Zhang, Jingya Liu, Lianhong Chen

**Affiliations:** College of Food Science and Technology, Southwest Minzu University, Chengdu, China

**Keywords:** hyperuricemia, probiotics, *Lactobacillus paracasei*, gut microbiota, uric acid, shortchain fatty acids

## Abstract

Hyperuricemia (HUA) is a metabolic disease arising from abnormal purine metabolism. It contributes to an increased risk of kidney damage. The present study aimed to investigate the uric acid (UA)-lowering effects of *Lactobacillus paracasei* 259 isolated from yak yogurt and explore its underlying mechanisms. Our results revealed that *L. paracasei* 259 decreased the UA levels in rats and inhibited the serum activities of xanthine oxidase. In addition, *L. paracasei* 259 reduced the levels of pro-inflammatory cytokines (tumor necrosis factor (TNF)-*α*, interleukin (IL)-1β, and IL-6) in the kidney and altered the expressions of UA transporters (ABC transporter 2 (ABCG2), PDZ domain containing 1 (PDZK1), urate transporter 1 (URAT1), and sodium-phosphate cotransporter type 4 (NPT4)) to near normal levels. Moreover, it increased the abundance of beneficial bacteria in the gut and recovered the gut microbiota composition, promoting the production of short-chain fatty acids (SCFAs). These findings suggested that *L. paracasei* 259 can potentially be used to decrease UA levels, repair kidney damage, regulate gut microbiota, and alleviate HUA.

## 1 Introduction

Hyperuricemia (HUA) is a metabolic disease arising from a long-term purine metabolism disorder (1). It is characterized by elevated serum uric acid (UA) levels, typically exceeding 420 μmol/L for men and 360 μmol/L for women (2). Several factors contribute to HUA development, such as genetics, high purine diet, renal insufficiency, kidney damage, obesity, and excessive alcoholic consumption (3). With improving living standards, HUA incidence has significantly increased worldwide (4). If not treated in a timely manner, HUA promotes UA deposition in the kidneys (5), thereby inducing gout, metabolic syndrome, atherosclerosis, hypertension, insulin resistance, and chronic kidney disease (6, 7).

To date, HUA is managed via three approaches, including drug therapy and biotherapy (2). Commercially available drugs such as allopurinol, febuxostat (8), and benzbromarone (9) have reportedly achieved good efficacy in HUA management. However, these drugs are also associated with several side effects, including liver and renal toxicities, rashes, diarrhea, and nausea (3). In this context, probiotic treatments confer several advantages, such as safety, efficiency, and fewer side effects. In humans, two-thirds of the UA in the body is excreted via the kidneys and the remaining is excreted via the intestine (10). Thus, modifying gut microbiota composition via probiotics is an attractive HUA management approach, especially when the kidneys are damaged (11). Previous studies have also shown that probiotics effectively promote purine breakdown and UA excretion (12).

Probiotics are active microorganisms that participate in metabolic activities and help maintain good health. In addition, they have been shown to efficiently recover gut microbiota composition (13). Furthermore, gut microbiota composition differs between the HUA-affected and -unaffected individuals, and probiotics can help revert the imbalance in the gut microbiota of the affected individuals (14). Probiotics promote short-chain fatty acid (SCFA) production by regulating gut microbiota, thereby inhibiting UA production, repairing the gut barrier, and inhibiting intestine translocation of harmful substances (15). In addition, probiotics can modify the expression levels of UA transporters in the intestine and during systematic inflammation response, both related to HUA development (16). Previous studies have reported that several strains of probiotics, such as *Lactobacillus plantarum* Q7 (17) and *L. brevis* DM9218 (18), exhibit promising potentials in reducing UA levels.

In this study, the UA-lowering activity of *L. paracasei* 259 was verified in an *in vivo* HUA rat model. In addition, we explored the potential mechanisms underlying HUA alleviation by investigating UA synthesis and excretion, pro-inflammatory cytokine levels, SCFA production, and gut microbiota composition. Our results could be used as a theoretical basis for the development of probiotics to manage HUA.

## 2 Materials and methods

### 2.1 Reagents

Potassium oxonate, adenine, and sodium carboxymethyl cellulose (CMC-Na) were purchased from Shanghai Macklin Biochemical Technology Co., Ltd. (China). Allopurinol was obtained from Hefei Julian Pharmaceutical Co. (China). UA, creatinine (CRE), blood urea nitrogen (BUN), and xanthine oxidase (XOD) were attained from Nanjing Jiancheng Technology Co., Ltd. (China). Tumor necrosis factor (TNF)-*α*, interleukin (1 L)-1β, and 1 L-6 were purchased from Mlbio (China). Total RNA isolation reagent, reverse transcription kit, and universal SYBR qPCR master mix were supplied by Biosharp (China). Methanol was provided by Merck (United States). Acetate, propionic acid, butyric acid, valeric acid, (3-dimethylamino-propyl)-ethyl-carbodiimide hydrochloride (EDC.HCl), and 2-nitrophenylhydrazine hydrochloride (NPH.HCl) were supplied by Aladdin (China). AxyPrep DNA gel extraction kit was obtained from Axygen Biosciences (USA).

### 2.2 Strain cultivation

Three hundred lactic acid bacteria strains were isolated from Sichuan Tibetan traditional fermented yak yogurt (Sichuan China) and stored in glycerin at −80°C. Of these strains, *L. paracasei* 259 exhibited efficient nucleoside degrading and xanthine oxidase inhibitory activities *in vitro*. Thus, 4% (*v/v*) *L. paracasei* 259 strains were inoculated in deMan Rogosa Sharpe (MRS) broth medium and cultured at 37°C for 24 h. After subculturing thrice, the fermentation broth was centrifuged at 8,000 rpm at 4°C for 15 min. The sediments were washed twice with 0.85% saline. Finally, varying concentrations of strain suspension were obtained after dilution with 0.85% saline.

### 2.3 Design of animal experiments

Specific pathogen-free (SPF) SD 48 rats (all 8-week-old males, with each weighing 200 ± 20 g) were purchased from Chengdu Dashuo Experimental Animal Co. (Animal Qualification Certificate Number: SCXK (Chuan) 2020–030). The rats were housed at 22 ± 2°C under a fixed 12-h artificial light period and a relative humidity of 60 ± 5%. All animal were performed humane care in strict accordance with the guidelines of the care and use of laboratory animals. The animal experiments study was reviewed and approved by the Animal Ethics Committee of Southwest Minzu University (Chengdu, China; approval number: SWUN-A-0067).

**Figure 1:**
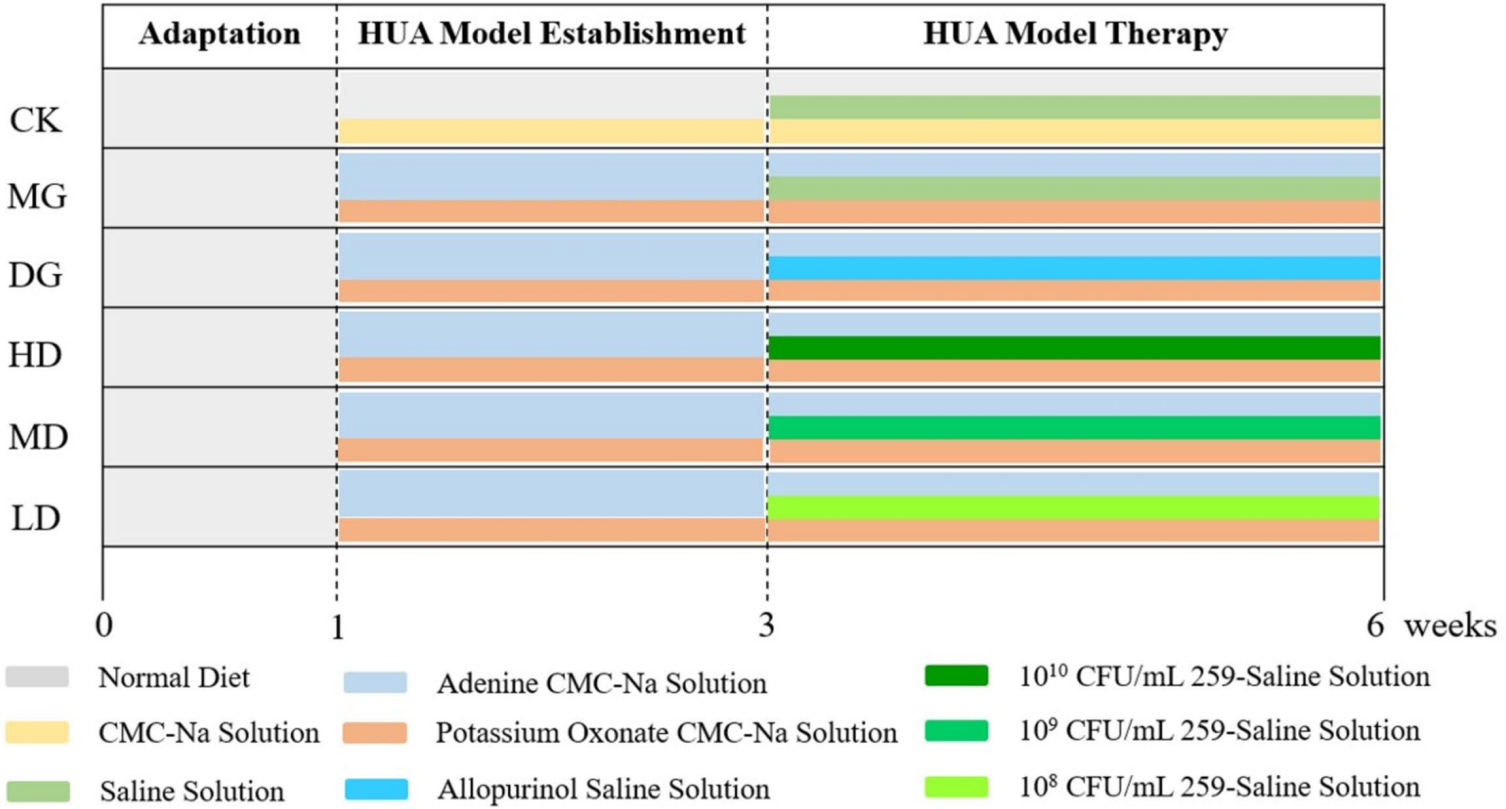
Design of animal experiments.

The design of the animal experiments is shown in Figure 1. All rats were acclimatized for 1 week before the experiments. Also, water and a standard chow diet were provided *ad libitum* at 24 h. Then, the rats were randomly divided into two groups. The rats in the control group (CK, *n* = 8) got a both injection and gavage with a 5% CMC-Na sterile solution at 9:00 am for 2 weeks. To establish the HUA model, the rats in the large model group (LMG, *n* = 40; each group 8 rats) were intraperitoneally injected with 300 mg/kg of potassium oxonate and nasogastric tube with 100 mg/kg of adenine at 9:00 am for 2 weeks.

Serum levels of UA, CRE, and BUN were significantly increased in LMG group rats, successful HUA induction, the LMG rats were randomly assigned to five groups: Model group (MG), drug treatment group (DG), and high (HD), medium (MD), and low (LD) bacterial suspension-treated groups (*n* = 8 for each group). The MG rates were administered with sterile saline via a gavage. The DG rats were nasogastric tube with 10 mg/kg of allopurinol dissolved in sterile saline. The HD, MD, and LD rats were nasogastric tube with 10^10^, 10^9^, and 10^8^ CFU/mL *L. paracasei* 259 strain suspension, respectively. All treatments were given at 3:00 pm for 3 weeks. Twelve hours after the last intervention, hepatic, renal, and intestinal tissues and intestinal contents were collected and stored at −80°C until further analysis.

### 2.4 Body weight, organ coefficient, and feed intake measurement

The body weights (M_1_), organ weights (M) and feed intake of the rats were monitored by electronic scale. Organ indices and organ indices augmenter of the rats were calculated based on their body and organ weights using the following formulae (19).


$$\mathrm{Index}\;1\;\left(\%\right)=\frac{M_{\mathrm{Kidney}}}{M_{1}}\times 100\%$$

$$\mathrm{Index}\;2\;\left(\%\right)=\frac{M_{\mathrm{Liver}}}{M_{1}}\times 100\%$$

$$\mathrm{Augmenter}\;1\;\left(\%\right)=\frac{MG_{\mathrm{kidney indexes}}-HD_{\mathrm{kidney indexes}}}{MG_{\mathrm{kidney indexes}}}\times 100\%$$

$$\mathrm{Augmenter}\;2\;\left(\%\right)=\frac{MG_{\mathrm{liver indexes}}-HD_{\mathrm{liver indexes}}}{MG_{\mathrm{liver indexes}}}\times 100\%$$

### 2.5 Serum diagnosis assay

Rat blood samples were collected from the tail veins on the 14th, 21st, and 42nd days. The blood samples were centrifuged for serum biochemical index detection. The serum levels of UA, BUN, CRE, and XOD were determined using respective kits.

### 2.6 Histopathological examination of the kidney

We performed a histopathological examination to evaluate the effects of bacterial suspension administration. Firstly, fresh kidney samples from all groups were rinsed with 0.85% sterile saline. Then, a small kidney fragment was cut and submerged in 4% paraformaldehyde solution for at least 24 h. Next, the tissues were subjected to alcohol dehydration and then embedded in paraffin. The samples were then cut into 4-μm thick slices. Finally, the slices were stained with hematoxylin and eosin, and observed under a microscope of 160x magnification (20).

### 2.7 Quantification of inflammation cytokines

The kidney samples were homogenized and suspended in 0.85% saline. Each homogenized mixture was centrifuged at 4,000 rpm for 20 min, and the supernatant was collected. The TNF-*α*, 1 L-1β, and 1 L-6 levels in the supernatants were quantified using kits. According to the manufacturer’s protocols, add supernatants and enzyme-labeled reagent to ELISA plate, 37°C incubate 60 min, clean 5 times, add color developing agent, 15 min in 37°C and dark place, termination reaction and 450 nm experiment.

### 2.8 Analysis of fecal SCFAs

Fecal SCFAs were analyzed by pre-derivatization high-performance liquid chromatography (PD-HPLC). Briefly, 0.15 g of fecal samples were homogeneously dispersed in 0.6 mL deionized water. Each mixture was centrifuged at 12,000 rpm for 10 min and the supernatant was collected. Each supernatant was treated with EDC.HCl and NPH.HCl. Then, the resultant solution was acidified with 42.5% phosphoric acid solution and extracted with diethyl ether. The resultant solution was dried up in nitrogen to obtain a solid residue. The residues were then dissolved in methyl alcohol and subjected to HPLC analysis (chromatographic column of Poroshell 120 EC-C18: 250 × 4.6 mm, 0.4 μm, 40°C, gradient elution method, with detection at 230 nm and final calculation based on the external standard quantitative method).

### 2.9 Reverse transcription-quantitative polymerase chain reaction (RT-qPCR) assay

The mRNA expression levels of ABC transporter 2 (ABCG2, in the intestine), PDZ domain containing 1 (PDZK1, in the intestine), urate transporter 1 (URAT1, in the kidney), and sodium-phosphate cotransporter type 4 (NPT4, in the kidney) were estimated using RT-qPCR. Briefly, total RNA was extracted from the kidney and intestinal samples by homogenizing in a total RNA isolation reagent, centrifuge and extract obtained. The total RNA was reverse transcribed to obtain cDNA by kit. Then, the cDNA samples were subjected to fluorescence quantification using the 3-step amplification method (2). The forward and reverse primers used for PCR are listed in Table 1. The 2^−△△Ct^ method was adopted to normalize the expression levels of the genes using *β*-actin as a reference gene.

**Table 1:** Primers used for PCR.

Gene	Forward (5′–3′)	Reverse (5′–3′)
URAT1	F: CATGTGAGGATGGCTGGGTT	R: GTGTCTGCCTCTGCCTTTCT
NPT4	F: GCCCTACAAGTGAGCAGTGT	R: GAGTCGGCTGCGTTCATTTG
ABCG2	F: GTAGGTCGGTGTGCGAGTCA	R: GGCCGTTCTTGTTTCTCTGTG
PDZK1	F: ACCCGACCTTGGGATGAATG	R: GAACACGCCCTTTTTACCTTGG
β-actin	F: CACGGCATTGTCACCAACTG	R: CCAGGAAGGAAGGCTGGAAG

URAT1, urate transporter 1; NPT4, sodium-phosphate cotransporter type 4; ABCG2, ABC transporter 2; PDZK1, PDZ domain containing 1.

### 2.10 16S rRNA gene sequencing

Genomic DNA were extracted from intestinal contents using the DNA extraction kit. Subsequently, the 16S rRNA gene were amplified from the samples using the forward primer 27F (AGRGTTYGATYMTGGCTCAG) and reverse primer 1492R (RGYTACCTTGTTACGACTT). PCR products were examined by the 2% agarose gel electrophoresis. The PCR products were purified using commercial AxyPrep DNA gel extraction kit according to the manufacturer’s instructions and quantified by QuantiFluor™ -ST blue fluorescence quantification system. Finally, a PCR amplicon was sequenced by Shanghai Biozeron personal biotechnology Co., Ltd. (China) after library construction.

### 2.11 Statistical analysis

Data were expressed as mean ± SD, and all analyses were performed in triplicate. ANOVA normality test and Duncan’s test were used for statistical analyses. Dormal distribution of data, *p* < 0.05 and *p* < 0.01 were deemed statically significant.

## 3 Results

### 3.1 Establishment of the HUA rat model

**Figure 2:**
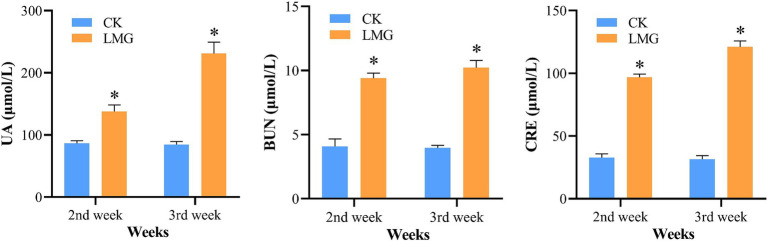
Analysis of serum levels of UA, BUN, and CRE. ^*^p < 0.05, compared with CK. UA, uric acid; BUN, blood urea nitrogen; CRE, creatinine.

To establish the HUA rat model, the LMG rats were intraperitoneally injected with 300 mg/kg of potassium oxonate dissolved in 5% CMC-Na sterile solution daily for 2 weeks. HUA development in rats was verified by quantifying the serum levels of UA, BUN, and CRE (Figure 2). Compared to the CK rats, the LMG rats exhibited significantly higher serum levels of UA, CRE, and BUN at weeks 2 and 3 post-experiment (*p* < 0.05), suggesting that the HUA model was successfully built.

### 3.2 Analysis of body weight, organ coefficient, and forage intake

As shown in Table 2, the initial weights of the CK and LMG rats were similar (*p* > 0.05). However, after HUA development, the body weights of both groups differed significantly (*p* < 0.05). Furthermore, compared to the MG rats, the remaining LMG rats exhibited significantly higher body weights (*p* < 0.05). In addition, the HD rats exhibited a higher total food intake than the MG rats, suggesting that *L. paracasei* 259 administration positively impacted food intake in rats. Furthermore, among all the groups, the MG rats exhibited the highest liver and kidney indexes (*p* < 0.05), with 29.07 and 24.18% higher indexes than the HD rats. Moreover, the liver index of the HD rats was comparable to that of the CK rats (*p* > 0.05). These findings indicated that *L. paracasei* 259 played a considerable role in HUA alleviation.

**Table 2:** Analysis of body weights, organ indexes, and food intakes of rats.

Group	Initial weight/g	3rd week weight/g	Final weight/g	Liver index/%	Kidney index/%	Food intake/g
CK	271.68 ± 6.54^a^	330.04 ± 8.19^a^	404.86 ± 7.27^a^	26.26 ± 0.69^e^	7.72 ± 0.39^d^	7512.70
LMG	268.86 ± 7.26^a^	272.74 ± 7.76^b^	/	/	/	/
MG	/	/	330.66 ± 5.90^e^	38.05 ± 1.29^a^	16.42 ± 0.82^a^	6338.40
DG	/	/	371.81 ± 8.96^c^	28.40 ± 0.43^cd^	12.43 ± 0.30^c^	7138.78
HD	/	/	393.66 ± 6.18^b^	26.99 ± 0.38^de^	12.45 ± 0.69^c^	7465.14
MD	/	/	368.19 ± 7.50^c^	29.24 ± 0.98^c^	13.52 ± 0.38^b^	7234.69
LD	/	/	342.59 ± 7.05^d^	32.71 ± 0.82^b^	15.61 ± 0.60^a^	6943.77

Lowercase letters indicate significant differences among the groups (*p* < 0.05). CK, control group; LMG, large model group; MG, model group; DG, drug treatment group; HD, group treated with the high concentration of bacterial suspension; MD, group treated with the medium concentration of bacterial suspension; LD, group treated with the low concentration of bacterial suspension.

### 3.3 Determination of serum biochemical indicators

**Figure 3:**
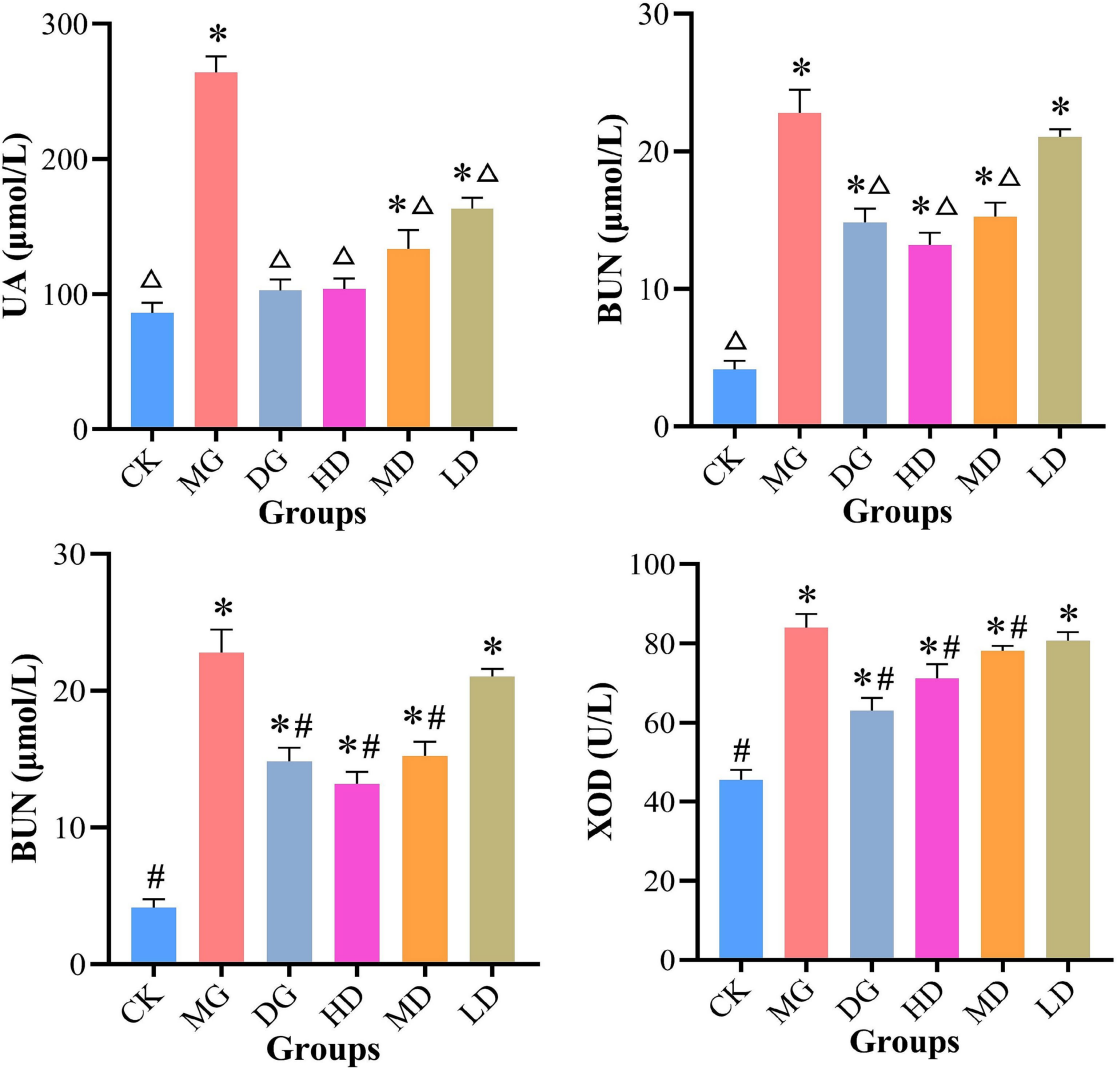
Analysis of serum biochemical indicators. ^△^*p* < 0.01, compared with MG; ^#^*p* < 0.05, compared with MG; ^*^p < 0.05, compared with CK. MG, model group; CK, control group.

Next, we investigated the effects of *L. paracasei* 259 administration by analyzing serum biochemical indicators in HUA-affected rats (Figure 3). After the treatments, the UA levels of HD and DG rats were significantly lower than MG groups (*p* < 0.01) but comparable to those of CK rats (*p* > 0.05). Furthermore, compared with MG, the HD, MD, and LD rats exhibited reduced CRE and BUN levels, with most prominent reduction observed for HD rats (35.88 and 42.08%, respectively). In addition, the XOD activities in HD and MD rats decreased as compared with MG. These results suggested that *L. paracasei* 259 administration significantly affected the serum biochemistry of HUA-affected rats.

### 3.4 Effects of *L. paracasei* 259 administration on kidney histopathology

**Figure 4:**
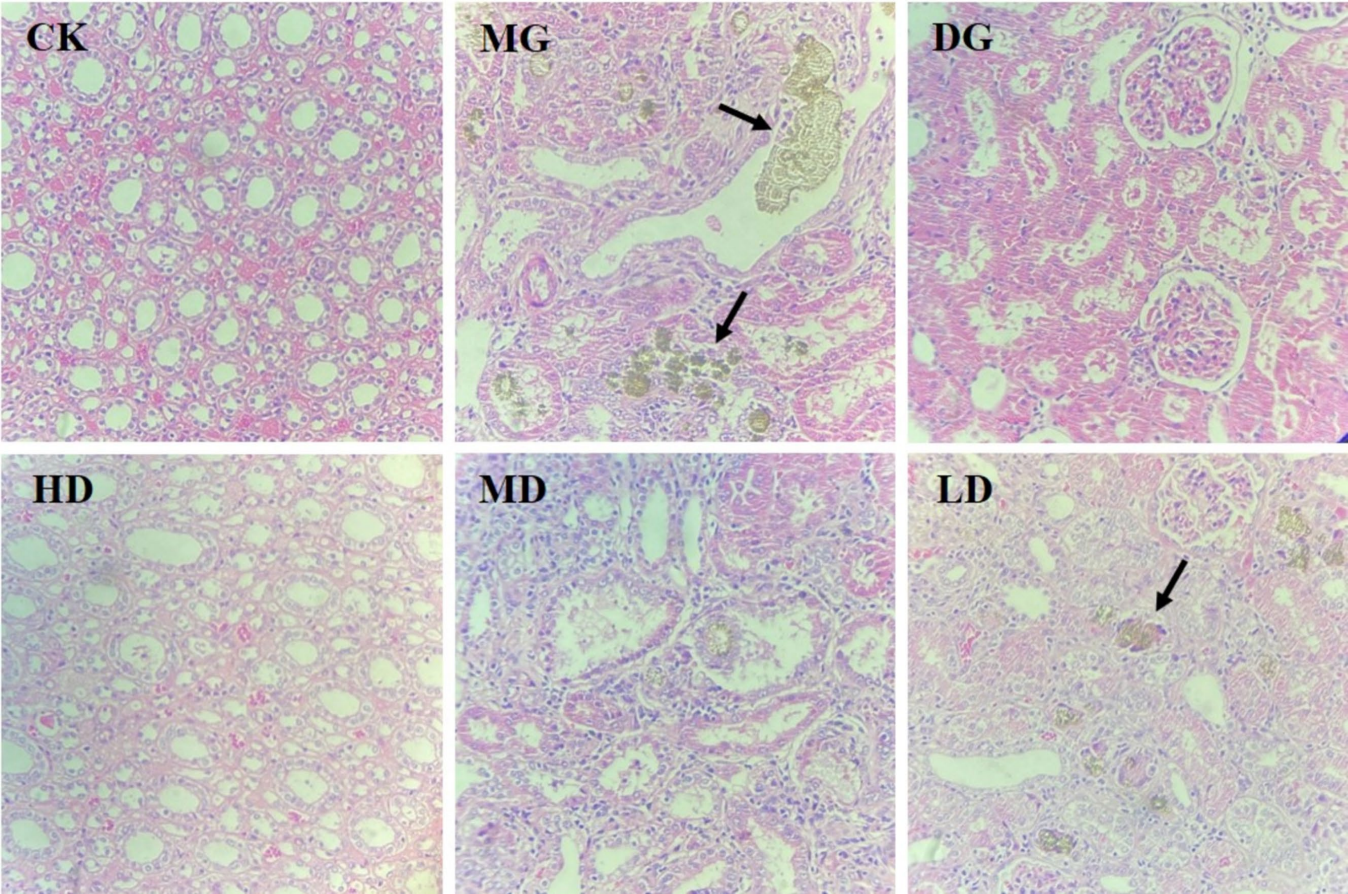
Hematoxylin and eosin staining of rat kidney tissue (magnification: 160x).

Next, we assessed the histopathology of rat kidney tissues to assess the protective effects of *L. paracasei* 259 on HUA-induced kidney damage. As shown in Figure 4, the kidney tissue of the CK rats exhibited normal structure and morphology, while that of the MG rats exhibited disorganized renal tubule arrangement, visible renal tubular atrophy, and deposition of brown urates in the renal tubular mesenchyme. After the treatments, the HD rats exhibited amelioration of HUA-induced kidney damage, neatly arranged renal tubule structure, and no significant urate deposition.

### 3.5 Quantification of inflammatory cytokines in rat kidney

**Figure 5:**
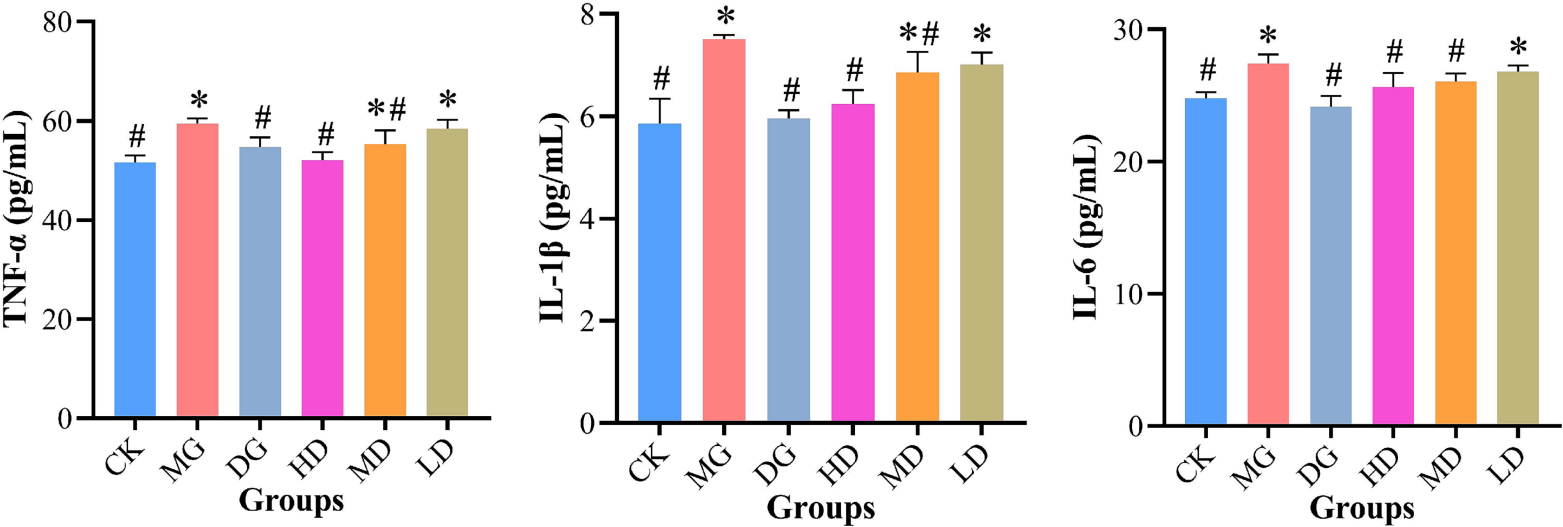
Analysis of levels of pro-inflammatory cytokines TNF-*α*, IL-1*β*, and IL-6 in rat kidney tissue. ^#^*p* < 0.05, compared with MG; ^*^p < 0.05, compared with CK. TNF-α, tumor necrosis factor-α; IL-1β, interleukin-1β; IL-6, interleukin-6; MG, model group; CK, control group.

Next, we quantified TNF-*α*, IL-1β, and IL-6 levels in the rat kidney to further evaluate the effects of *L. paracasei* 259 (Figure 5). After the treatments, the MG rats exhibited higher TNF-*α*, IL-1β, and IL-6 levels than the remaining LMG groups. Notably, the IL-1β levels of the HD rats were significantly lower than MG groups (*p* < 0.05) and were comparable to those of CK rats (*p* > 0.05). Furthermore, the CK, HD, and DG rats exhibited comparable TNF-α and IL-6 levels. Taken together, the HUA-induced production of pro-inflammatory cytokines in rat kidneys were effectively alleviated by *L. paracasei* 259 administration.

### 3.6 Effects of *L. paracasei* 259 administration on fecal SCFAs

**Figure 6:**
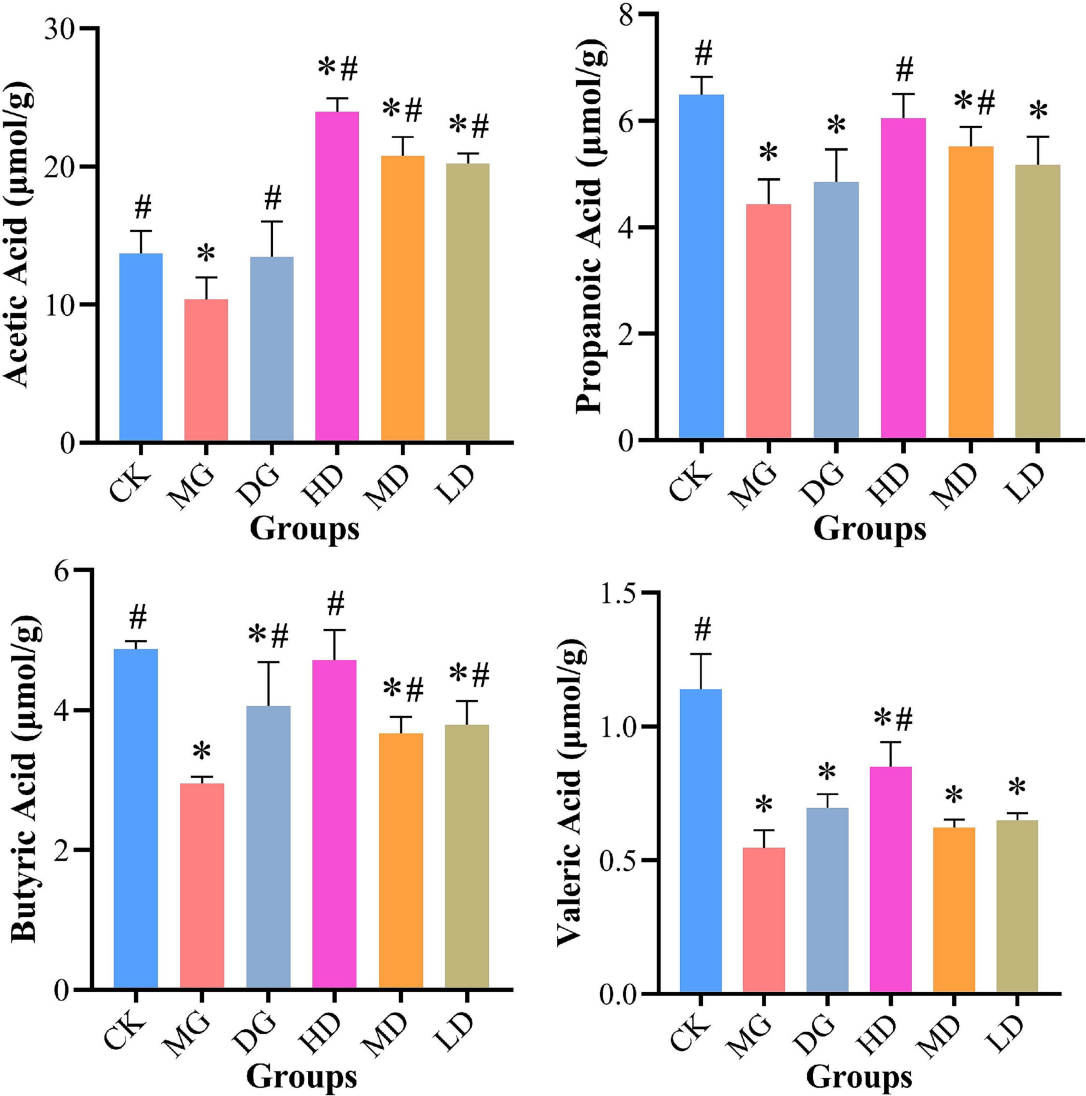
Effects of *Lactobacillus paracasei* 259 administration on fecal SCFAs. ^#^*p* < 0.05, compared with MG; ^*^p < 0.05, compared with CK. SCFAs, short-chain fatty acids; MG, model group; CK, control group.

The fecal SCFA levels were measured using PD-HPLC (Figure 6). After the treatments, we observed an improvement in HUA-impacted fecal SCFA levels in *L. paracasei* 259-administered rats. For instance, the fecal samples from the HD, MD, and LD rats exhibited significantly higher acetic acid levels than the samples from the CK rats (*p* < 0.05). The propanoic acid and butyric acid levels in the samples from the HD rats were significantly higher (by 36.32 and 59.71%, respectively) than those in the samples from the MG rats. Compared with CK rats, the propanoic acid of MG group was decreased by 31.74%. Furthermore, the samples from the HD rats exhibited higher valeric acid levels (0.85 ± 0.09 μmol/g) among all groups. These results demonstrated that *L. paracasei* 259 impacted fecal SCFA levels.

### 3.7 Expression levels of UA transporters in rat kidney and intestine

**Figure 7:**
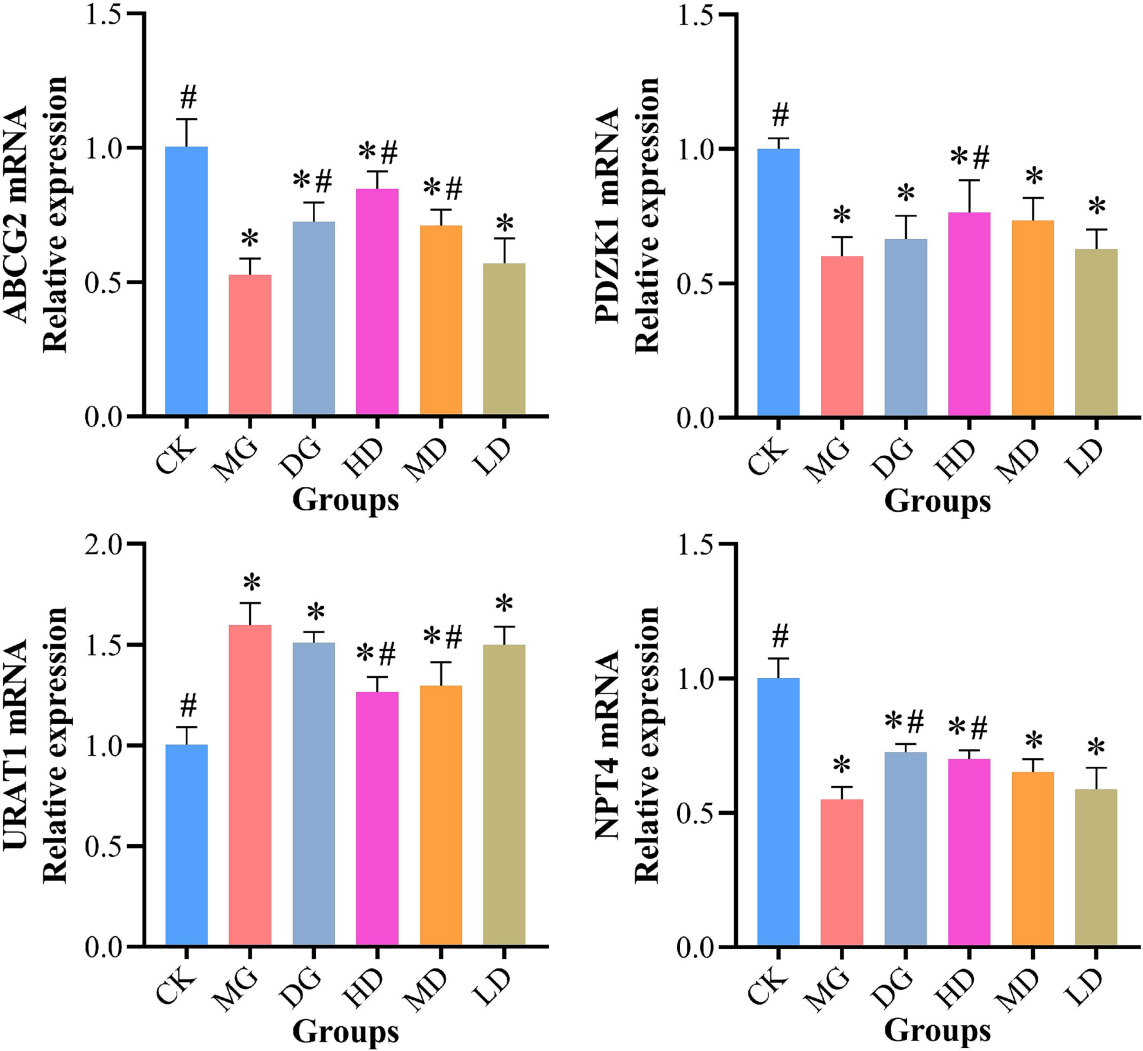
Effects of *Lactobacillus paracasei* 259 administration on the UA transporter levels in rat kidney and intestine. ^#^*p* < 0.05, compared with MG; ^*^p < 0.05, compared with CK. Note: UA, uric acid; MG, model group; CK, control group.

We used RT-qPCR to assess the effects of *L. paracasei* 259 on UA transporters in HUA-affected rats. As shown in Figure 7, the HD rats exhibited significantly higher intestinal ABCG2 and PDZK1 levels than the MG rats (*p* < 0.05). Moreover, compared with MG, the *L. paracasei* 259-administered rats exhibited decreased kidney URAT1 levels, with most prominent decrease observed for the HD rats (by 20.85%). In addition, the kidney NPT4 levels moderately recovered after *L. paracasei* 259 administration as compared with MG. These results suggested that *L. paracasei* 259 markedly impacted the levels of UA transporters in HUA-affected rats.

### 3.8 Effects of *L. paracasei* 259 administration on the gut microbiota of rats

**Figure 8:**
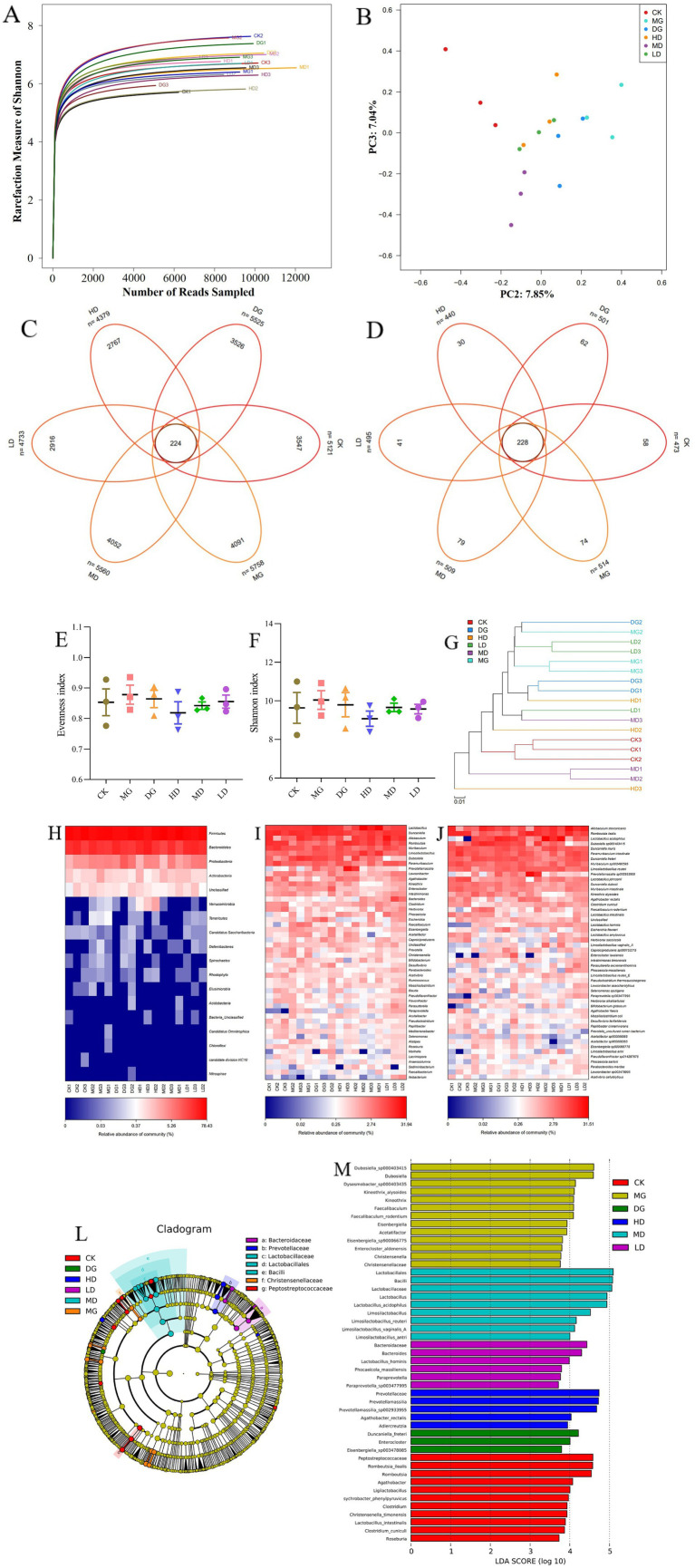
Effects of *Lactobacillus paracasei* 259 administration on the gut microbiota of the HUA-affected rats. **(A)** Shannon rarefaction curves of fecal samples. **(B)** Gut microbiota β-diversity. **(C)** Venn diagram depicting OTU distribution. **(D)** Venn diagram depicting species distribution. **(E, F)** Evenness and Shannon indexes. **(G)** Cluster tree analysis of no weighted unifrac. **(H–J)** Heat map analysis of bacteria abundances at the phylum, genus, and species levels. **(K)** Cluster tree analysis based on LEfSe results. **(L)** LDA score of the LEfSe results. HUA, hyperuricemia; OTU, operational taxonomic unit; LDA, linear discriminant analysis; LEfSe, Linear discriminant analysis Effect Size.

Next, we used 16S rRNA sequencing to investigate the effects of *L. paracasei* 259 on the gut microbiota of HUA-affected rats. As sequencing depth increased, the Shannon rarefaction curves gradually flattened, indicating that our sequencing results efficiently reflected the abundance and variety of our samples (Figure 8A). Two Venn diagrams depicted the numbers of operational taxonomic units (OTUs) and species across the groups and exclusive to each group (Figures 8C,D). Our results indicated that the gut microbiota varied across different LMG groups. We examined and compared the *α*-diversities of the microbial communities across all groups (Figures 8E,F). We observed mild differences in the Evenness and Shannon indexes across the groups. In addition, the *β*-diversities of the gut microbiota of all groups were investigated using principal coordinates analysis (PCoA) (Figure 8B) and observed marked intergroup differences. Furthermore, the HD and MD groups were relatively close to the CK group in the cluster tree, whereas the MG group was relatively far (Figure 8G).

Meanwhile, the relative bacterial abundances were also evaluated at the phylum, genus, and species levels. As illustrated in Figure 8H, we detected higher abundances of Bacteroidetes and Firmicutes in the HD and MD groups than in the MG group. The MG rats exhibited high abundances of Elusimicrobia, Spirochaetes, Rhodophyta, Tenericutes, Deferribacteres, and Chloroflexi; however, these phyla were almost absent in the HD, MD, and CK groups. At genus level, the MG group exhibited lower abundances of *Limosilactobacillus*, *Romboutsia*, *Faecalibaculum*, *Bifidobacterium*, and *Lactobacillus* and higher abundances of *Prevotella*, *Eisenbergiella*, *Desulfovibrio*, and *Acetatifactor* than the remaining LMG groups (Figure 8I). At species level, the HD and MD groups exhibited higher abundances of *L. johnsonii*, *Limosilactobacillus reuteri*, *L. acidophilus*, and *Li. antri* than the MG group (Figure 8J). The Linear discriminant analysis Effect Size (LEfSe) results (Figures 8K,L) showed high abundances *of Christensenella*, *Eisenbergiella*, and *Christensenellaceae* among the dominant genera in the MG group. Interestingly, high abundances of *Prevotellamassilia*, *Lactobacillus*, and *Limosilactobacillus* were detected in the HD and MD groups. Overall, these results suggested that the gut microbiota of HUA-affected rats were moderately recovered by *L. paracasei* 259 administration.

## 4 Discussion

HUA is a metabolic disease arising from abnormal elevation in the blood UA levels. It might lead to urarthritis and can cause a plethora of diseases, such as hypertension, hyperlipidemia, insulin resistance, non-alcoholic fatty liver disease, and cardia-cerebrovascular disease (21, 22). Numerous drugs have been synthesized to treat HUA; however, they are associated with serious adverse reactions. Thus, probiotics-based therapies have gained extensive attention due to their negligible side effect (23). The current study investigated the effects of *L. paracasei* 259 on HUA management. We observed a reduction in the serum levels of UA, BUN, CRE, and XOD in the HUA-affected rats, which corroborated the findings of Wu et al. (24). In addition, the HUA-affected rats that were administered with the bacterial suspension exhibited weight gain, decreased liver and kidney indexes, and recovered food intake, which was consistent with the results of Cao group (23). Xiao et al. also reported that lactic acid bacteria aided in the recovery of tubular structures and kidney glomerulus in HUA-affected rats (25). These findings indicated that *L. paracasei* 259 could potentially be used for HUA management.

Excessive UA leads to the production of pro-inflammatory cytokines (26). Previous studies have shown that *Lacticaseibacillus rhamnosus* Fmb14 decreases TNF-*α* and IL-1β levels (27). Sun et al. (28) also found that *L. paracasei* decreased TNF-α, IL-1β, and IL-6 levels. These findings suggested that *L. paracasei* 259 can be used for efficient HUA management in rats. PDZK1 and ABCG2 play important roles as UA transporters (29) and are mainly expressed in the small intestine (30). Chen et al. (31) found that PDZK1 and ABCG2 are simultaneously regulated, and they substantiated the interaction between these transporters. In the current study, the expressions of PDZK1 and ABCG2 decreased in the MG group, but they normalized after bacterial administration (32). The kidney is main organ of UA excretion. Therefore, we also explored the effects of *L. paracasei* 259 on UA transporters in the kidney. We found that *L. paracasei* 259 administration led to decreased URAT1 levels and increased NPT4 levels in HUA-affected rats. Similar studies have shown that altering the expressions of UA transporters could ameliorate UA excretion in rats (2). These results suggested that *L. paracasei* 259 could ameliorate HUA manifestations in rats.

The gut microbiota is a key factor influencing HUA-related morbidity, promoting UA excretion via SCFA production (33). Thus, the diversities in the gut microbiota could be detected by assessing intestinal SCFA levels (34). HUA alters gut microbiota composition by decreasing the abundance of beneficial bacteria and impacting gut microbiota diversity (35). Interestingly, we observed that the SCFA levels of HUA-affected rats recovered after *L. paracasei* 259 administration (36). Previous studies also demonstrated that probiotics can regulate gut microbiota composition in the HUA-affected rats (37). We observed that *L. paracasei* 259 restored the gut microbiota composition of HUA-affected rats with a high efficiency. The abundance of beneficial bacteria in the gut microbiota were higher in the HD group than in the MG group, suggesting that probiotics can be used to restore the gut microbiota of HUA-affected rats (38). Analysis of the *β*-diversity of the gut microbiota of HUA-affected rats (39) also showed that *L. paracasei* 259 administration effectively restored the gut microbiota of HUA-affected rats.

At phylum level, *L. paracasei* 259 administration in HUA-affected rats was found to reduce the abundances of Rhodophyta, Tenericutes, Chloroflexi, and Deferribacteres, and increase the abundances of Bacteroidetes and Firmicutes to near normal levels (23). Previous studies have shown that *Faecalibaculum* spp. promote SCFA production, alleviating HUA progression (40). In the current study, at the genus level, the *L. paracasei* 259-administered rats exhibited high abundances of *Faecalibaculum*, *Limosilactobacillus*, *Bifidobacterium*, and *Lactobacillus*. At the species level, these groups exhibited high levels of *L. johnsonii*, *Li. reuteri*, *Li. antri*, and *L. acidophilus*. Our findings were consistent with the results of previous studies showing that the HUA manifestations in rats can be ameliorated by suppressing inflammatory reactions and recovering gut microbiota composition (41). In the present study, the LEfSe results also showed that the impact of HUA emergence on the abundance of beneficial bacteria in the gut microbiota of rats was reversed after *L. paracasei* 259 administration. These results suggested that *L. paracasei* 259 can be used to manage HUA in rats by restoring gut microbiota.

## 5 Conclusion

*L. paracasei* 259 from traditional fermented yak yogurt exhibited excellent UA-lowering ability in *in vivo*, restoring kidney damage and downregulating serum biochemical indices. Moreover, *L. paracasei* 259 suppressed the inflammatory reactions in the kidney of HUA-affected rats and restored expressions of UA transporters to near normal levels. In addition, *L. paracasei* 259 also ameliorated the HUA-induced imbalance in the gut microbiota and promoted SCFA production. These findings suggested that *L. paracasei* 259 could potentially be used as a probiotic regimen to alleviate HUA.

## Data Availability

The data presented in the study are deposited in the NCBI repository, accession number PRJNA1130059. The data link: https://www.ncbi.nlm.nih.gov/bioproject/PRJNA1130059/.
